# Role of Anifrolumab in Refractory Cutaneous Manifestations of Lupus Erythematosus: A Case Series and Literature Review

**DOI:** 10.7759/cureus.39553

**Published:** 2023-05-27

**Authors:** Muhammad Atif Khan, Faiza Humayun Khan, Hina Benish Khan, Constantine Saadeh, Nichole Davey

**Affiliations:** 1 Internal Medicine, University of Kansas Medical Center, Kansas City, USA; 2 Internal Medicine, Khyber Medical University, Peshawar, PAK; 3 Physiology, Khyber Medical College, Peshawar, PAK; 4 Rheumatology, Allergy ARTS Clinic, Amarillo, USA

**Keywords:** systemic lupus erythematosus, cutaneous lupus erythematosus, discoid lupus erythematosus (dle), subacute cutaneous lupus erythematosus, anifrolumab

## Abstract

Lupus erythematosus (LE) is an autoimmune disease that presents either as a systemic (SLE) or an isolated skin disease (CLE). Currently, there is no FDA-approved medication specifically for CLE, and is treated with the same approach as SLE. We present two refractory cases of SLE with severe cutaneous manifestations unresponsive to the first-line therapy treated with anifrolumab. First, a 39-year-old Caucasian female with a known history of SLE with severe subacute CLE presented to the clinic for her refractory cutaneous symptoms. Her current regimen was hydroxychloroquine (HCQ), mycophenolate mofetil (MMF), and s/c belimumab with no improvement. Belimumab was discontinued, and she was started on anifrolumab with significant improvement. Another, a 28-year-old female with no known medical history was referred to a rheumatology clinic for elevated anti-nuclear antibody (ANA) and ribonucleoprotein (RNP) titers. She was diagnosed with SLE, and was treated with HCQ, belimumab, and MMF but failed to produce a reasonably good outcome. Hence belimumab was discontinued and anifrolumab was added instead with significant cutaneous improvement. The treatment spectrum for SLE is wide, which includes antimalarial (HCQ), oral corticosteroids (OCS), and immunosuppressants (Methotrexate-MTX, MMF, azathioprine-AZT). Anifrolumab, a type 1 IFNα receptor subunit 1 (IFNAR1) inhibitor, has been recently approved by the FDA for moderate to severe SLE while on standard therapy in August 2021. Early use of anifrolumab in moderate to severe cutaneous manifestations of SLE or CLE may result in significant improvement in patients.

## Introduction

Lupus erythematosus (LE) is an inflammatory autoimmune disease with multisystemic (systemic lupus erythematosus, SLE) or isolated skin (cutaneous lupus erythematosus, CLE) involvement. SLE has been on the rise with a prevalence of 20-150 cases per 100,000 worldwide [1]. Cutaneous lesions manifest in almost 70% of all SLE [2]. Lupus skin manifestations are further divided into subtypes depending upon the clinical features, histological changes, and serological abnormalities: 1) Acute CLE (ACLE); 2) Subacute CLE (SCLE); 3) Chronic CLE (CCLE), including discoid LE (DLE), chilblain LE (CHLE) and LE panniculitis (LEP); 4) intermittent CLE (ICLE). The severity of SLE is calculated by using the Cutaneous Lupus Erythematosus Disease Area and Severity Index (CLASI) [3].

Presently, no medication has been FDA-approved for the treatment of CLE specifically [2]. However, numerous therapies exist for the treatment of SLE ranging from preventive to topical and systemic therapies [2]. Antimalarials and corticosteroids are recommended as a first-line systemic treatment for SLE or CLE [4]. Patients develop an increased risk of scarring, disfigurement, and poor quality of life due to resistance to treatments. Biologic agents are studied for their efficacy in SLE. Anifrolumab and Belimumab have been approved for SLE only, not isolated skin disease [5]. Anifrolumab, a human monoclonal antibody, is a type-1 interferon (IFN) receptor subunit 1 blocker and has shown efficacy in adults with moderate to severe SLE [6]. We present two refractory cases of SLE with severe cutaneous manifestations treated with anifrolumab.

## Case presentation

Case 1

**Figure 1:**
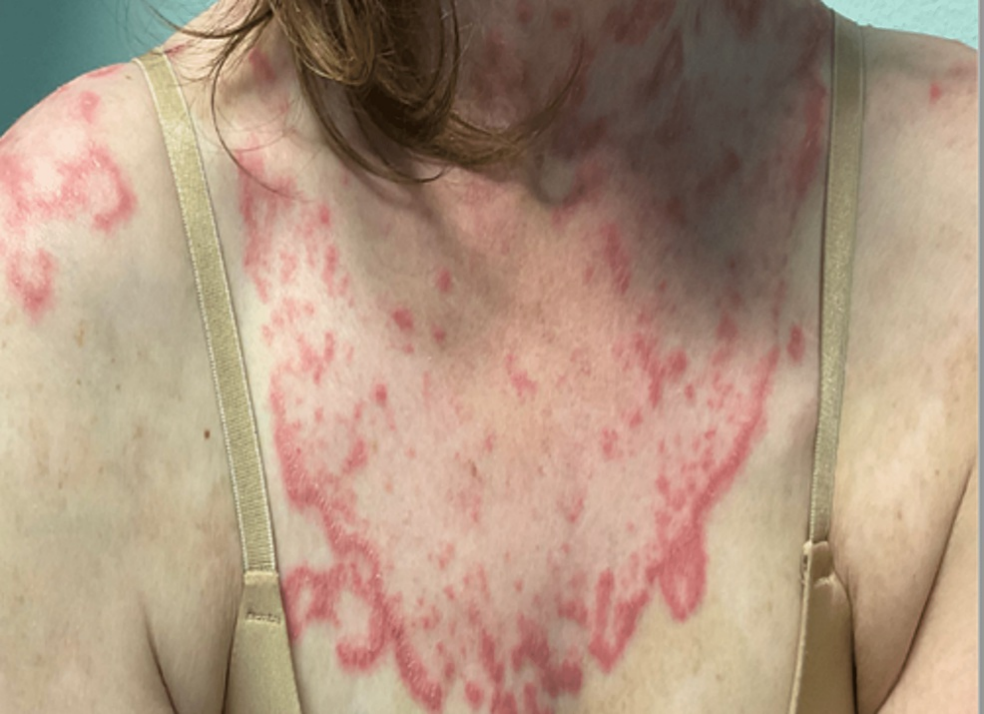
Case 1 - Cutaneous lesions on the chest before the use of anifrolumab

**Figure 2:**
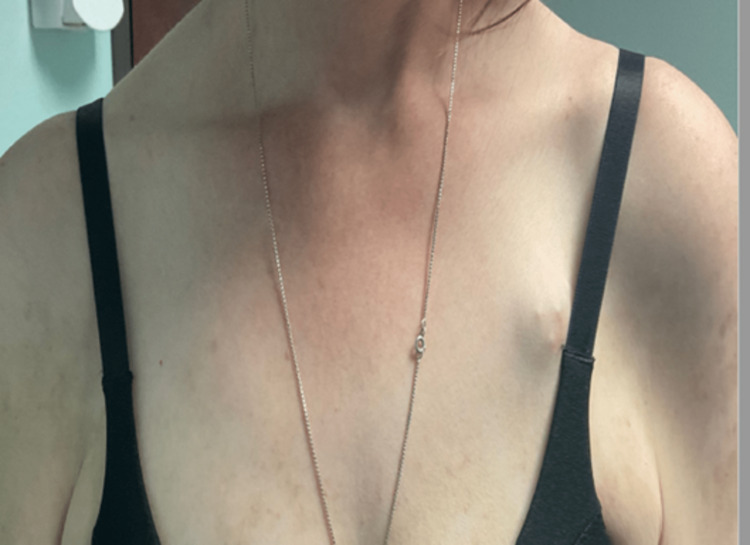
Case 1 - Cutaneous lesions on the chest after the use of anifrolumab

A 39-year-old Caucasian female with a known history of SLE for 18 years presented to the clinic for her refractory SCLE. She had intermittent flares of cutaneous lesions during this period. She also reported a remote history of lupus nephritis and enteritis. She failed to respond to cyclophosphamide, thalidomide, IV belimumab and was currently on hydroxychloroquine (HCQ), mycophenolate mofetil (MMF), and s/c belimumab. On examination, she had an annular erythematous lesion with a central scale on the upper extremities, anterior chest wall, back, and right lower leg with a malar rash (Figure 1). Her initial lab work is mentioned in Table 1. She did receive steroids as well prior to establishing care with us and was prescribed repository corticotropin (Acthar) injection for almost a year in the past as a steroid-sparing agent which did not improve her symptoms. As she did not respond we decided to start treatment with non-B cell therapy. Belimumab was discontinued, and she was started on intravenous (IV) anifrolumab 300mg every four weeks along with HCQ and MMF. The patient also received prednisone 60mg along with anifrolumab. No side effects were observed. Significant improvement in her cutaneous lesions was noticed in five months (Figure 2). Her leukopenia improved from 2.5 to 5.7 x 10^3^.

**Table 1:** Lab work on the initial visit of case 1. ANA=anti-nuclear antibody, dsDNA=anti-double-stranded deoxyribonucleic acid antibody, SM=anti-Smith antibody, C3=complement component 3, C4=complement component 4, RF=rheumatoid factor, ESR=erythrocyte sedimentation rate, CRP=C-reactive protein

Labs	Case 1	Reference Range
WBC	2.5	4.0-11.0 x 10^3^
ANA	156.9	<1.0 IU/ml
dsDNA	<9.8	<30.0 IU/ml
SM	3.4	<1.0 CU
C3	92.4	75-175 mg/dl
C4	19.6	22-45 mg/dl
RF-IgM	0.9	<60 IU/ml
ESR	8	<20 mm/hr
CRP	0.0	<1.0 mg/dl

Case 2

**Figure 3:**
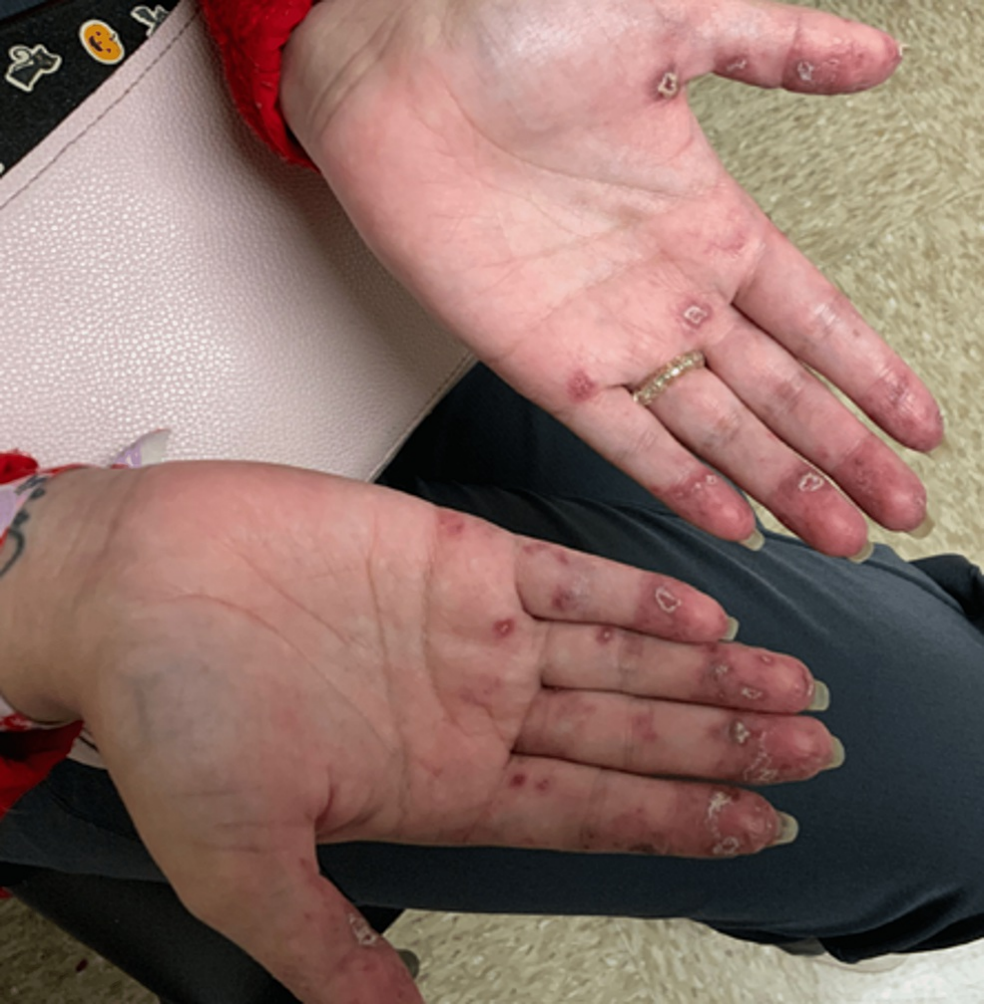
Case 2 - Cutaneous lesions on the hands before the use of anifrolumab

**Figure 4:**
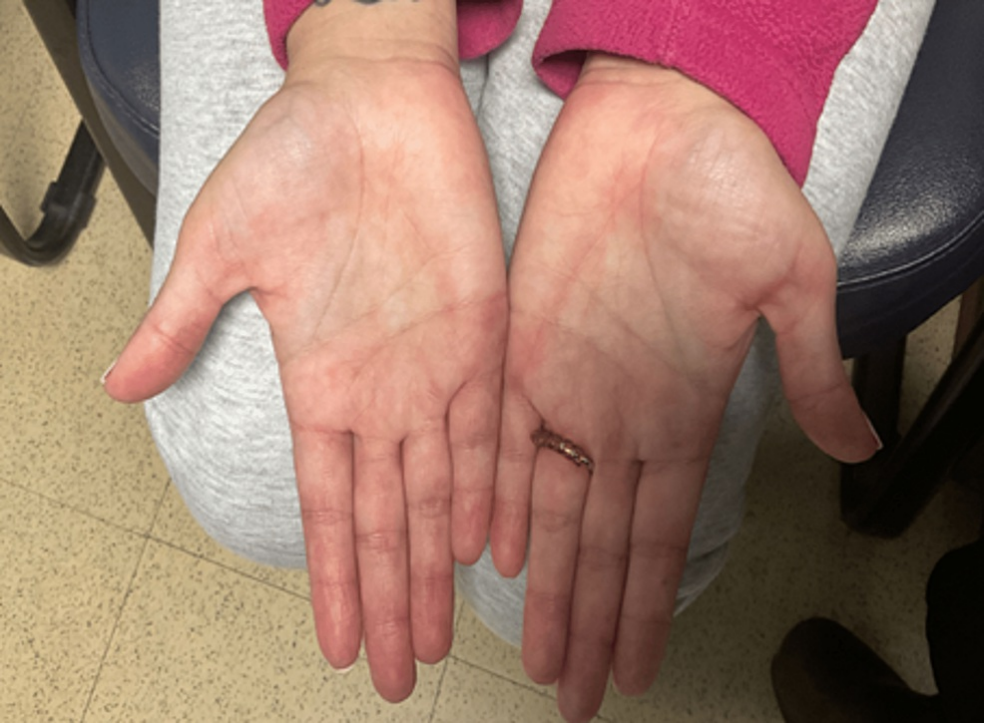
Case 2 - Cutaneous lesions on the hands after the use of anifrolumab

A 28-year-old female with no known prior medical history was referred to a rheumatology clinic for elevated anti-nuclear antibody (ANA) and ribonucleoprotein (RNP) titers. She complained of fatigue, polyarthralgia and intermittent rash for one year. On examination, she had a polycyclic erythematous rash on her face and bilateral hands (Figure 3). She had low C3, C4 complement and elevated anti-RO 60, anti-RNP, and anti-SM antibodies. Her initial lab work is mentioned in Table 2. She was diagnosed with SLE and started on HCQ. Belimumab, dapsone, and MMF were later added due to no response to HCQ. Noticing the persistence of cutaneous disease, belimumab was discontinued and IV anifrolumab 300 mg every four weeks was added to the regimen. No side effects were observed. She was able to taper prednisone 60mg to 30mg within three months of starting anifrolumab. Significant improvement was noticed in her cutaneous lesions in two months (Figure 4).

**Table 2:** Lab work on the initial visit of case 2. ANA=anti-nuclear antibody, dsDNA=anti-double-stranded deoxyribonucleic acid antibody, SM=anti-Smith antibody, C3=complement component 3, C4=complement component 4, RF=rheumatoid factor, ESR=erythrocyte sedimentation rate, CRP=C-reactive protein, Anti-RNP= antinuclear ribonucleoprotein.

Labs	Case 2	Reference Range
WBC	5.7	4.0-11.0 x 10^3^
ANA	165.2	<1.0 IU/ml
dsDNA	216.9	<30.0 IU/ml
Anti-SM	693.5	<1.0 CU
C3	67.0	75-175 mg/dl
C4	<6.72	22-45 mg/dl
RF-IgM	-	<60 IU/ml
ESR	64	<20 mm/hr
CRP	1.0	<1.0 mg/dl
Anti-RO 60	38	<20 CU
Anti- RNP	643.8	<1.0 U

## Discussion

The current standard of care treatment for SLE includes antimalarial (HCQ), oral corticosteroids (OCS), immunosuppressants (MTX, MMF, AZT), and biological agents (belimumab) [4]. Cyclophosphamide and rituximab are used in organ or life-threatening conditions [4]. Topical agents and dapsone, retinoids are used for skin manifestations [4]. Most of the medications used in SLE are also being used for isolated skin disease (CLE). On similar lines, our patients were treated as well. The treatment spectrum is wide, ranging from preventive measures including sun protection, smoking cessation, elimination of photosensitizing drugs, and vitamin D supplementation to topical and systemic therapies. Topical steroids are considered as first-line agents, and calcineurin inhibitors are used as steroid-sparing agents [7]. Antimalarial including hydroxychloroquine is recommended as first-line systemic therapy, which was also reported by Yokogawa et al. who found a significant reduction in symptom severity measured by CLASI [8,9]. Immunosuppressants, MTX and MMF, are reported to have efficacy as MTX is used as a 2nd line agent in refractory SCLE and DLE [10]. However, none of those medications, alone or in combination, resulted in the improvement of cutaneous lesions in our patients. Thalidomide and lenalidomide have not been studied yet in randomized control trials for their role in cutaneous lesions but some case reports have been published.

As we become more aware of the pathways involved in SLE, biological agents are now the focus of studies. The only approved biological agent by the FDA before the approval of anifrolumab was belimumab, a B-lymphocyte stimulator-specific inhibitor [5]. Both our patients were initially treated with belimumab with no satisfactory results.

Interferon pathway and its significance

Cytokines, including interferons (IFN), appear to play a major role in SLE. There are several types (type I, Ⅱ and type Ⅲ IFNs) however we will focus on type I IFN, producing IFNα, IFNβ and others. Type I IFNα receptor is the target for all type I IFN cytokines. IFNα is formed mainly by plasmacytoid dendritic cells (pDC) as a result of endosomal toll-like receptor interaction with nucleic acids while IFNβ is produced by almost any cell. A plasmacytoid dendritic cell is a rare cell population but has a unique capacity of producing high levels of IFNα [11]. IFNα binds to its target receptor and signals via JAK/STAT pathways leading to the transcription of genes, known as IFN signature (IFNGS) [12]. The outcome of IFNα stimulation results in the increased survival and activation of dendritic, B, and T cells [13]. High IFN levels have been demonstrated in patients with active SLE correlating with lupus activity index and with the titer of anti-dsDNA antibodies [14]. IFNα has been particularly associated with active mucocutaneous inflammation [14]. Similar results were shown by Postal et al. in childhood-onset SLE and elevated IFN-α in CSF of SLE retinopathy by Kondo et al. [15,16]. Genetic factors have played a role in increasing the susceptibility to develop SLE. For instance, TNFα-induced protein 3 (TNFAIP3) gene variants resulting in increased IFNα levels lead to increased susceptibility to develop SLE and single immunoglobulin IL-1-related receptor (SIGIRR) gene polymorphism has been associated with SLE in the Chinese population [17,18]. Due to its critical role in pathogenesis in SLE, the IFN pathway was used as a target for therapy.

Anifrolumab, a type 1 IFNα receptor subunit 1 (IFNAR1) inhibitor, has been approved by FDA for the use of moderate to severe SLE while on standard therapy in 2021. It also results in the internalization of IFNAR1, leading to reduced levels available for binding to cytokine [19]. Inhibition of the IFN signaling pathway results in blockage of IFNGS and downstream inflammatory and immunologic processes [19]. Its approval was based on the efficacy and safety data from the two TULIP phase 3 trials (TULIP1 & TULIP2) and the MUSE phase 2b trial. In the MUSE trial, the primary endpoint of a composite of SLE Responder Index [SRI (4)] response with sustained OCS reduction at Week 24 was met by more patients receiving anifrolumab (34.3% and 28.8% for patients receiving 300 mg and 1000 mg, respectively) than placebo (17.6%) [19]. The percentage of patients with a baseline CLASI activity score of ≥10 who had a ≥50% reduction in this score by week 52 was greater for both anifrolumab dosages (63.0% for 300 mg and 58.3% for 1,000 mg) compared with placebo (30.8%) [20]. After the failure of the TULIP 1 trial to meet primary endpoints, using the secondary endpoint as the primary endpoint, TULIP 2 trial showed that BICLA (British Isles Lupus Assessment Group [BILAG]-based Composite Lupus Assessment) response at 52 weeks occurred in 47.8% of patients receiving anifrolumab and 31.5% receiving placebo. Among patients with active skin disease (CLASI ≥10) at baseline, a reduction of 50% or more in the CLASI at week 12 occurred in 49.0% of the patients receiving anifrolumab as compared to 25.0% receiving placebo [6]. Considering the data from trials and its recent approval for SLE, anifrolumab was used as a last resort for our patients and resulted in a dramatic improvement in cutaneous lesions.

## Conclusions

Cutaneous manifestations and the resulting permanent disfigurement in some cases can be of significant distress to the patients. Anifrolumab, a recent FDA-approved drug for systemic lupus, has been associated with significant improvement in the refractory cutaneous manifestations of SLE and can potentially be used early in the disease course in patients with moderate to severe cutaneous manifestations.
